# Empowerment Weekends for young adults

**DOI:** 10.1186/1750-1172-9-S1-O35

**Published:** 2014-11-11

**Authors:** Annette Lemli, Nicole Schwarzer

**Affiliations:** 1 SoMA e.V., Munich, 80997, Germany

## 

SoMA is the German patient organization for people with an anorectal malformation (children born without an anus or with an anus ending in a wrong location) and Hirschsprung’s disease. Anorectal malformations may be associated with further malformations (e.g. bladder, sexual organs, spine, or cardiac defects) and the patients often face lifelong problems such as incontinence. Because of the lack of medical centres and as physicians for adults, who do not know much about the treatment, there are special problems during transition. Due to legal retention periods for medical reports a retrospective access is often impossible. But precise medical reports are of high importance, and the patients should know their correct diagnosis and what kind of surgery they went through.

For that reason SoMA in 2009 started with the project Empowerment Weekend (seminar “My own medical file”) for a maximum of eight teenagers between 14 and 25 years. They bring their personal medical reports and in a one-to-one discussion a paediatric surgeon looks through the reports and explains to the patient the individual malformation. They look for possible gaps in the diagnosis, the follow-up is discussed, and further examinations may be recommended. There is an individual psychological counselling, a lecture by the paediatric surgeon, a nurse shows different medical devices for the follow-up, there is a pedagogical offer under the direction of the SoMA social worker, and time for some leisure programme. After the weekend each participant has an own folder with all the medical files, and he or she understands the malformation and is in a position to explain it to others.

SoMA has evaluated the first three seminars, and the youngsters indicated an increase in their self-esteem and that they now have a better knowledge of their malformation. Mistakes concerning the diagnosis were also discovered during the seminars

In 2011 the seminars got awarded the “Care prize” from a private German health insurance company, and from ACHSE, the German National Alliance for chronic rare diseases.

## Conclusions

The Empowerment Weekends for young adults show how important it is that especially patients with a rare disease understand their malformation, take over responsibility, and care for themselves. Medical reports should be given to the parents and patients, and the diagnosis should be explained to them in detail.

The project is transferable to other rare diseases. It is crucial to involve patient organizations in the organization of those projects.

